# Molar‐Scale Phenolic Acid Decarboxylation Using Thermostable Biocatalysts and Enzyme‐Compatible Deep Eutectic Solvents

**DOI:** 10.1002/cssc.202501755

**Published:** 2025-11-09

**Authors:** Sonja Vaupel, Lars‐Erik Meyer, Pablo Domínguez de María, Selin Kara

**Affiliations:** ^1^ Institute of Technical Chemistry Leibniz University Hannover Callinstr. 5 30167 Hannover Germany; ^2^ Sustainable Momentum, SL. Av. Ansite 3, 4‐6 Las Palmas de Gran Canaria 35011 Canary Islands Spain; ^3^ Biocatalysis and Bioprocessing Group Department of Biological and Chemical Engineering Aarhus University Gustav Wieds Vej 10 8000 Aarhus C Denmark

**Keywords:** deep eutectic solvents, kinetics, phenolic acid decarboxylases, process intensification, thermostability

## Abstract

The synthesis of biogenic hydroxy styrenes by enzymatic decarboxylation of phenolic acids (e.g., ferulic, *p*‐coumaric, caffeic, or sinapic acids) is a promising route involving biorefineries and biocatalysis, in which the low substrate solubility in water requires the quest for nonconventional media. This work explores the tunability of deep eutectic solvents (DES) to simultaneously create enzyme‐compatible and solubilizing systems, in which thermostable phenolic acid decarboxylases (PAD) may operate at 50–70 °C, decreasing the inherent DES viscosity. Four DESs displaying high solubility of ferulic acid are assessed (with additions of up to 20 vol% of phosphate buffer): Choline chloride–glycerol (ChCl–Gly, 1:2), choline chloride–ethylene glycol (ChCl–EG, 1:2), choline acetate–glycerol (ChAc–Gly, 1:2), and betaine–glycerol (Bet–Gly, 1:2). Bet–Gly DES exerts promising properties, being a halide‐free solvent, an enzyme‐stabilizer at 50–70 °C (1.4 higher melting temperatures for the N31 ancestor *Bacillus subtilis* PAD), and 14‐fold higher ferulic acid solubility compared to buffer. Intensified conditions of up to 1 M ferulic acid (≈200 g L^−1^, in a slurry‐like system) in Bet‐Gly (1:2, 20 vol% buffer) lead to excellent conversions (90%) in less than 5 h, demonstrating that biocatalysis can be performed under industrially relevant conditions when DES media are tuned for the specific requirements of the application.

## Graphical abstract

Thermostable phenolic acid decarboxylases (PADs) catalyze the synthesis of hydroxy styrenes in deep eutectic solvents (DESs) tuned for solubility and enzyme stability. Betaine–glycerol (1:2) is halide‐free, stabilizing PADs at 50–70 °C, and increases ferulic acid solubility 14× over buffer. Up to 90% conversion of 1 M ferulic acid in <5 h demonstrates DESs' potential for biocatalysis.

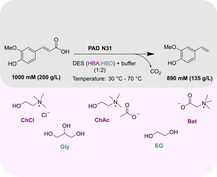

## 1 Introduction

The intensification of enzyme‐catalyzed processes to meet industrial requirements with sufficient substrate loadings (≥100 g L^−1^) is crucial for the efficient valorization of (biobased) chemicals,^[^ ^1^ ^]^ as high substrate loadings and product titers reduce the costs associated with downstream processing and make the overall process more economical and environmentally friendly. Moreover, these higher substrate concentrations enable an efficient use of reactor volume, facilitating scale‐up. All these considerations have consequently sparked a debate on using water as a reaction medium, as most industrially relevant compounds exhibit low water solubility.^[^ ^2^, ^3^ ^]^

In biorefineries, enzymes enable a sustainable shift to biobased polymers through advances in protein and process design.^[^ ^4^, ^5^, ^6^, ^7^, ^8^, ^9^, ^10^, ^11^, ^12^, ^13^, ^14^, ^15^, ^16^ ^]^ Enzymatic decarboxylation of biogenic phenolic acids (e.g., ferulic, *p*‐coumaric, caffeic, sinapic) offers a promising way to valorize lignocellulosic residues as alternatives to petrochemical feedstocks.^[^ ^17^, ^18^, ^19^ ^]^ The decarboxylated products, typically hydroxystyrenes, can serve as versatile intermediates in polymer chemistry, as cross‐linking agents in adhesive formulations, as comonomers to tailor polymer properties, or as dimerized antioxidants.^[^ ^20^, ^21^ ^]^ The decarboxylation is catalyzed by phenolic acid decarboxylases (PADs), homodimeric, cofactor‐free enzymes belonging to lyases (EC 4), which decarboxylate hydroxycinnamic acids such as caffeic acid, *p*‐coumaric acid, and ferulic acid (FA), affording the corresponding vinyl‐phenols. Recently, Myrtollari et al. (2024) identified a thermally stable ancestor of PAD from *Bacillus subtilis* (*Bs*PAD, PDB entry: 2P8G) using ancestral sequence reconstruction (ASR). The original sequences were reconstructed by inferring a phylogenetic relationship between modern homologs and applying a statistical model of amino acid substitution to calculate sequences at internal nodes of the phylogenetic tree. As a result, a thermally stable ancestor (PAD N31, PDB: 8B30), with an elevated unfolding temperature of 78.1 °C (20 °C higher than wild‐type *Bs*PAD) and a half‐life of 45 h (<1 min for *Bs*PAD) at 60 °C, was reported.^[^ ^22^, ^23^ ^]^

When developing industrial biocatalysis, trade‐offs aligning the optimal reaction parameters and the enzyme requirements are needed, in particular for the selection of a suitable solvent.^[^ ^24^ ^]^ Enzymes generally demand aqueous media, while many relevant organic substrates are poorly soluble in water.^[^ ^4^, ^25^ ^]^ The decarboxylation of the phenolic acids is particularly challenging (solubilities of ≈0.92 g L^−1^ for ferulic acid or ≈1.23 g L^−1^ for caffeic acid in water at room temperature),^[^ ^26^ ^]^ limiting the productivity of the decarboxylation reaction.^[^ ^27^ ^]^ To achieve higher substrate loadings, enzymatic reactions in nonconventional media (e.g., solvent‐free, organic solvents, supercritical fluids, ionic liquids, or deep eutectic solvents (DESs)) can be advantageous.^[^ ^3^, ^28^, ^29^ ^]^ A pioneering approach was introduced by Pesci et al. (2017), disclosing a biphasic system of hexane and phosphate buffer to intensify the decarboxylation, reporting 89% conversion of ferulic acid at 100 mM (19 g L^−1^).^[^ ^30^ ^]^ Building upon this foundation, our group further optimized the reaction in organic media. Petermeier et al. (2024) demonstrated the conversion of substrate concentrations of 2 M (400 g L^−1^) ferulic acid using “water‐saturated” cyclopentyl methyl ether (CPME), catalyzed by wild‐type *Bs*PAD immobilized on amino carriers.^[^ ^31^ ^]^ PAD catalysis in “wet” CPME was upscaled to full conversion of 630 mM ferulic acid in a 10 L rotating bed reactor, leading to 288 g/L/d of volumetric productivity.^[^ ^32^ ^]^

As an alternative to more classic organic media, the use of DESs can be considered. DESs are formed by the interaction of hydrogen bond donors (HBDs) and hydrogen bond acceptors (HBAs) with a wide range of composition options, creating a broad (tunable) palette of solvents that can combine enzyme‐compatibility with high solubilization and biogenic origin.^[^ ^33^, ^34^, ^35^ ^]^ The application of DES for biocatalysis has been extensively explored in terms of i) enzyme activity, ii) thermal stability, and iii) substrate solubility.^[^ ^34^, ^36^ ^]^ So far, oxidoreductases,^[^ ^37^, ^38^, ^39^, ^40^, ^41^, ^42^, ^43^ ^]^ transferases,^[^ ^44^, ^45^ ^]^ hydrolases,^[^ ^46^, ^47^, ^48^, ^49^, ^50^ ^]^ and lyases^[^ ^51^, ^52^, ^53^ ^]^ have been applied in DESs. Furthermore, the use of DESs to enhance substrate solubility has been documented, e.g., dimethyl‐3‐phenylglutarate with a 27‐fold increased solubility in 50 vol% ChCl–urea,^[^ ^54^ ^]^ daiadazin with a 1.5‐fold in choline chloride–ethylene glycol (ChCl–EG) (2:1),^[^ ^55^ ^]^ or gelose starch with ≈37 g in 100 g in betaine–urea.^[^ ^56^ ^]^

Ecological and safety‐wise advantages have been claimed for DES, such as high thermal stability, low volatility, nonflammability, and biodegradability, although more research related to the fate of used DES (incineration or dilution to wastewater treatment plants)^[^ ^57^ ^]^ and the potential of microorganisms to upcycle them is still needed.^[^ ^58^ ^]^ For a practical application, a frequent challenge is the high viscosity of DESs (e.g., ≈235 mPa·s for ChCl:Gly (1:2) compared to water, ≈0.89 mPa·s, both at room temperature).^[^ ^27^, ^34^, ^59^ ^]^ Lowering high viscosities can be achieved by adding water as cosolvent, and/or using higher temperatures.^[^ ^47^ ^]^ The latter approach would enable the setup of processes with less water content (higher solubility capacity), but would require thermostable enzymes. Such combinations of engineered proteins with tailored solvents would be a powerful synergy for future biorefineries, creating “media‐agnostic biocatalysts” to be adapted to perform reactions in realistic crude broths.^[^ ^60^ ^]^

Concerning intensified processes in DES, our group recently reported the highest biocatalytic conversion with complete conversion of 800 mM (≈100 g L^−1^) cinnamaldehyde to cinnamyl alcohol via horse liver alcohol dehydrogenase (HLADH) catalysis in hydrophobic DES (lidocaine–oleic acid, 80 vol%, 20 °C).^[^ ^41^ ^]^ For PAD catalysis, Schweiger et al. (2019) explored the utilization of DESs, achieving 77% conversion of 400 mM (≈72 g L^−1^) caffeic acid within 24 h using 50 vol% ChCl–Gly (1:2) with the wild‐type *Bs*PAD.^[^ ^51^ ^]^ In parallel, Myrtollari et al. (2024) demonstrated a further improvement in conversion by achieving 80% conversion of 400 mM (≈77 g L^−1^) ferulic acid in 70 vol% ChCl–Gly (1:2) at 60 °C.^[^ ^22^ ^]^ Looking for future synergies, the use of the recently discovered, thermally stable PAD in conjunction with tailored DESs at higher reaction temperatures could be envisioned. This system would: i) reduce the viscosity of DES by combining some water addition and higher temperature, ii) increase substrate solubility by carefully tailoring the DES media, iii) enhance the activity of the enzyme by designing compatible systems, and iv) enable the introduction of halide‐free DESs, which could have more manageable waste treatment.^[^ ^57^ ^]^

**Scheme 1:**
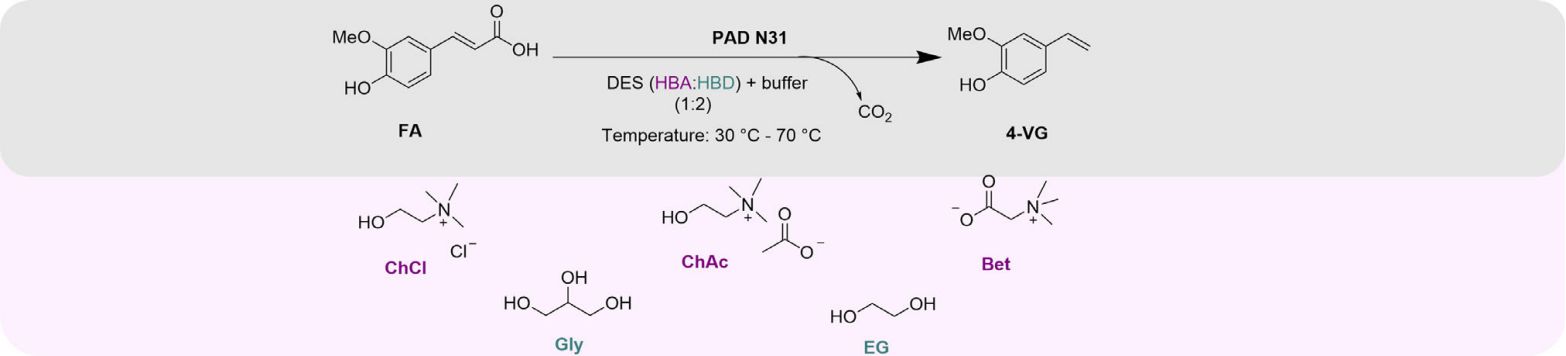
Intensification of the ferulic acid decarboxylation catalyzed by phenolic acid decarboxylase N31 (PAD N31) in designed DES–buffer (50 mM KPi, pH 6) mixtures at 50 °C. HBA: hydrogen bond acceptor, HBD: hydrogen bond donor. Bet: Betaine, ChCl: choline chloride, ChAc: choline acetate, EG: ethylene glycol, Gly: glycerol; FA: ferulic acid, and 4‐VG: 4‐vinylguaiacol.

With those considerations in mind, this work describes the intensification of the (thermostable) PAD N31‐catalyzed decarboxylation of ferulic acid in DES‐water media. For this work, the specific focus was on: i) the solubility of ferulic acid in different DESs, ii) the temperature and water‐content‐dependent viscosity of the designed DESs, iii) the activity of PAD N31 in the new media at high temperature, iv) the stability of PAD N31, and v) the intensification of the process through high substrate loadings (**Scheme** **1**).

## 2 Results and Discussion

Glycerol‐based DESs were selected based on the positive contribution of the HBD glycerol to the enzyme's stability.^[^ ^37^ ^]^ The molar ratio was chosen based on the ferulic acid solubility in choline chloride and betaine (*N*,*N*,*N*‐trimethylammonioacetate)‐based DESs.^[^ ^61^ ^]^ DES were characterized with respect to the ferulic acid solubility and to the viscosity related to temperature and water content. The PAD N31´s enzyme‐catalyzed reaction was assessed in terms of activity, stability, and optimal operational temperature. By selecting a promising case, the enzymatic reaction was intensified to industrial substrate loadings.

### 2.1 Characterization of the DES Solvent System

#### 2.1.1 Ferulic Acid Solubility

**Figure 1:**
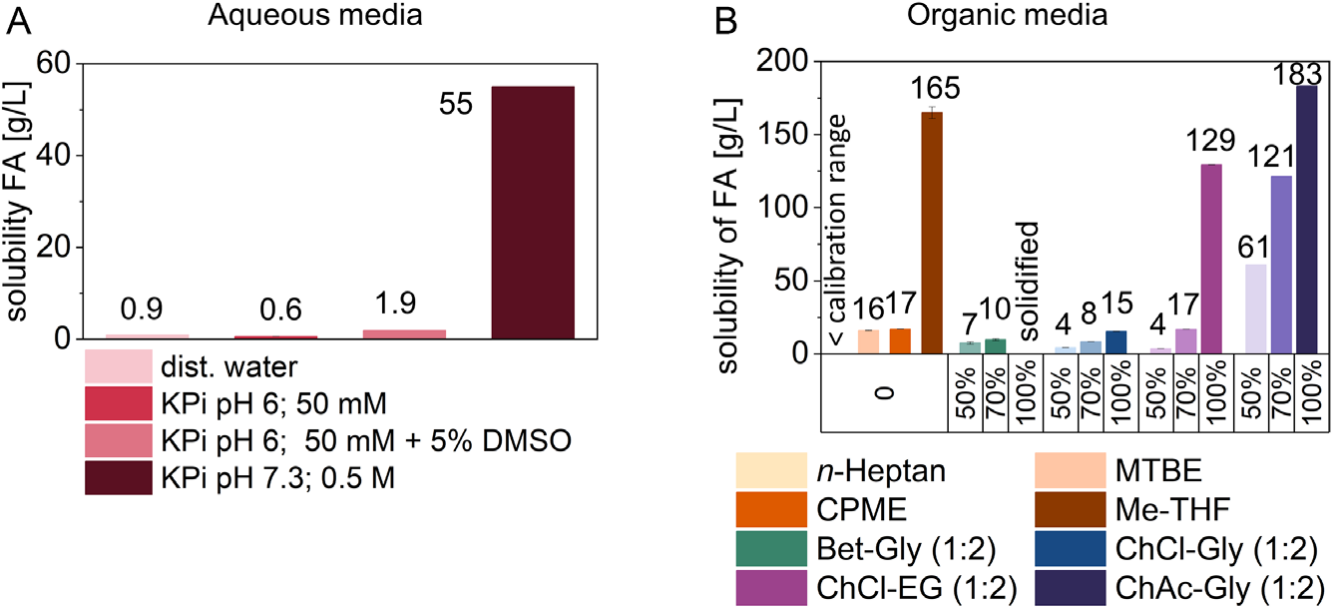
The solubility of ferulic acid (FA) in various solvent systems at 30 °C: A) aqueous media and B) organic media and DES‐based systems. KPi =  Potassium phosphate buffer, DMSO =  dimethyl sulfoxide, MTBE =  *tert*‐methyl‐butyl ether, CPME =  cyclopentyl methyl ether; 2–MeTHF = 2‐methyltetrahydrofuran, Bet =  betaine, Gly =  glycerol, ChCl =  choline chloride, EG =  ethylene glycol, ChAc =  choline acetate. All solubilities determined in aqueous media and organic solvents are adapted from Petermeier et al. (2024).^[^ ^26^ ^]^

Ferulic acid exerts low solubilities in pure water (4.6 mM, 0.90 g L^−1^) or in buffer solutions (50 mM KPi, pH 6, being 3.2 mM, 0.62 g L^−1^) at 30 °C.^[^ ^26^ ^]^ By adding a cosolvent (e.g., dimethyl sulfoxide (DMSO), 5 vol%), it increases threefold to 9.8 mM (1.9 g L^−1^). The FA solubility improves in aqueous conditions at alkaline pH by a simultaneous tenfold expansion of the buffer capacity (500 mM KPi, pH 7.5) to 283 mM (55 g L^−1^) at 30 °C (**Figure** **1**A), presumably due to the deprotonation in the slightly alkaline environment. However, although the initial pH is within the enzyme's operational range, the decarboxylation of the acidic substrate triggers an increase in the pH, hindering the conversion process.^[^ ^26^ ^]^ Titration with a base may be an alternative, yet it adds complexity to the system.^[^ ^30^ ^]^

Previously, our group investigated the FA solubility in organic solvents considered to be enzyme‐compatible,^[^ ^26^ ^]^ such as methyl‐*tert*‐butyl ether (MTBE), cyclopentyl methyl ether (CPME), 2‐methyltetrahydrofuran (2‐MeTHF), and *n*‐heptane (Figure 1B, first four entries). FA was poorly soluble in *n*‐heptane (below the calibration limit), while MTBE and CPME dissolved FA up to 82 mM (16 g L^−1^) and 88 mM (17 g L^−1^) at 30 °C, respectively. 2‐MeTHF showed a higher FA solubility, yet poor enzyme conversions were observed. Overall, FA is more soluble in polar organic solvents than in apolar ones.^[^ ^26^ ^]^ Of the solvents screened, water‐saturated CPME proved to be the most promising candidate for enzymatic decarboxylations.^[^ ^26^, ^31^, ^32^ ^]^

**Figure 2:**
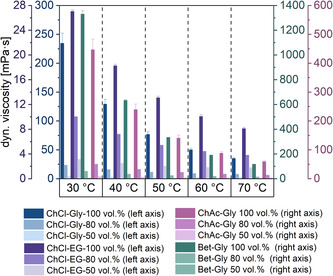
The viscosity of DESs and DES–water mixtures determined by rheometric measurements. Viscosity was measured for increasing shear rates for 1–1000 1/s at temperatures of 30 °C to 70 °C. All measurements were carried out in duplicates. Buffer (50 mM KPi, pH 6) represents the water added to prepare DES–water formulations.

Since FA solubility increases in polar organic media, the chosen hydrophilic DESs showed an overall high solubility as well (Figure 1B, last four entries).^[^ ^22^, ^34^, ^61^, ^62^ ^]^ This can be explained by the formation of hydrogen bonds by FA with its functional groups (–OH and –COOH), reflected in a better solubility at higher DES contents (50, 70, and 100 vol%). For the two glycerol‐based DESs, ChCl–Gly (1:2) and Bet–Gly (1:2), solubilities were 43 mM (≈8.4 g L^−1^) and 50 mM (≈9.7 g L^−1^), respectively (at 70 vol% DES with buffer (50 mM KPi, pH 6)). The used HBAs—ChCl and Bet—can stabilize FA with their cationic structure and prevent precipitation, yet the HBD glycerol can form strong hydrogen bonds with FA, increasing the viscosity, and making solute–solvent interactions less frequent and effective. In Bet–Gly (at 100% DES), FA solidified and could not be resolubilized even at increased temperatures. Conversely, ChCl–Gly displayed a fourfold improvement in the FA solubility from 50 vol% to 100 vol%, whereas ChCl–EG (1:2) exhibited the second‐highest solubility at 664 mM (129 g L^−1^), which declined to 87 mM (≈16.8 g L^−1^) and 19 mM (≈3.7 g L^−1^) with the addition of 30 vol% (70 vol% DES) and 50 vol% buffer (50 mM KPi, pH 6, 50 vol% DES), respectively. The enhanced solubility observed when using EG can be attributed to the lower viscosity, which allows for molecular mobility while still forming hydrogen bonds (**Figure** **2**).^[^ ^61^ ^]^ The highest solubility was observed in ChAc–Gly (1:2), reaching a dissolved concentration of FA of 943 mM (≈183 g L^−1^). Upon the addition of 30 vol% buffer (50 mM KPi, pH 6, 70 vol% DES), the solubility decreased by 34%. The best candidates for process intensification were ChCl–EG (1:2) and ChAc–Gly (1:2), with 208‐fold and 295‐fold increase in FA solubility, respectively. However, other effects should be assessed for an enzymatic reaction, not only the solubilizing effect (see below). Moreover, a compromise between substrate solubility (by H‐interaction with the DES components) and substrate availability for the biocatalysts needs to be found, to assure not only a proper dissolution, but an efficient reaction system as well.

#### 2.1.2 Viscosity of DESs: Role of Temperature and Water Content

High DES contents are needed for high FA solubility, resulting in an increased viscosity. To reduce these impractical high viscosities, buffer/aqueous solutions can be added^[^ ^47^ ^]^ or a higher temperature applied. 100 vol% Bet–Gly (1:2) exhibited the highest dynamic viscosity at 30 °C (1331 mPa·s), followed by ChAc–Gly (1:2) (447 mPa·s), ChCl–Gly (1:2) (235 mPa·s, 30 °C), and ChCl–EG (1:2) (27 mPa·s, 30 °C). The viscosity of Bet–Gly (1:2) could be lowered to 60 mPa·s by adding 20 vol% buffer (50 mM KPi, pH 6), and further reduced to 5.7 mPa·s at 50 vol% DES at 30 °C. For ChAc–Gly (1:2), the viscosity was reduced by 99% by the addition of 50 vol% buffer (50 mM KPi, pH 6), (Figure 2). The addition of 20 vol% buffer to ChCl–Gly (1:2) (80 vol%) resulted in a 90% reduction in viscosity. Similarly, the viscosity drops from 235 mPa·s (100 vol% DES, 30 °C) to 23 mPa·s (80 vol% DES) and 4.3 mPa·s (50 vol%). The lowest viscosity was observed for ChCl–EG (27 mPa·s). Chloride anions tend to engage with water molecules in the same geometric arrangement as they do with choline or glycerol. Therefore, due to the numerical superiority of water molecules, they can capture a substantial number of chloride anions even with relatively low water content. This leads to an exponential decline in the number of choline cations or glycerol molecules that depend solely on chloride for interconnecting.^[^ ^63^ ^]^

Likewise, higher temperatures displayed a less pronounced effect on viscosity than that observed for the water addition. For example, the viscosity of Bet–Gly (1:2) decreased from 1331 to 333 mPa·s by raising the temperature from 30 to 50 °C. Analogously, the viscosity of ChCl–Gly (1:2) could be lowered by 67% when the temperature was raised from 30 to 50 °C. ChAc–Gly (1:2) shows a decline of 70% from 30 to 50 °C with viscosities of 447 mPa·s at 30 °C, 141 mPa·s at 50 °C, and 59 mPa·s at 70 °C. This trend is also observed for ChCl–EG, with a viscosity of 27 mPa·s at 30 °C, declining to 18.3 mPa·s at 40 °C, 13 mPa·s at 50 °C, 10 mPa·s at 60 °C, and finally to 8 mPa·s at 70 °C.

Overall, the DESs’ viscosity can be significantly reduced by buffer addition and temperature increase, and trends can be assumed based on the nature of the DESs. Abbott and coworkers discovered the “hole theory”, which links the presence of holes in a liquid to the mobility of compounds.^[^ ^64^ ^]^ They proposed that volumetric factors, including steric effects, affect viscosity more than intermolecular interactions between HBAs and HBDs. The void size distribution depends on the type of HBA and HBD. This theory explains the decreasing viscosity as temperature rises. At lower temperatures, the voids are small compared to the DES components, restricting movement and increasing viscosity. At higher temperatures, void sizes match DES components, enhancing mobility. For instance, adding a hydroxyl group increases viscosity significantly.^[^ ^65^ ^]^

The viscosity data suggest that ChCl–Gly and ChCl–EG are the suitable candidates for the reaction with viscosities of 235 and 27 mPa·s at 30 °C, respectively. Higher temperatures would be favorable for the reaction system due to lowered viscosities. However, the enzyme may suffer from temperature‐induced deactivation or the DES’ nature, and these aspects were therefore investigated subsequently, to find an adequate trade‐off for the reaction media.

### 2.2 Characterization of the Thermostable PAD in the DESs

#### 2.2.1 Activity of PAD N31 in Different DES–Buffer Mixtures

The activity of PAD N31 in each DES with different water contents (ranging from 70 to 90 vol%), as well as the conversion of FA after 24 h at 30 °C, was determined (**Table** **1**, Figure S1, Supporting Information).

The enzymatic activity decreased with increasing DES content. For ChAc–Gly, very low activities in the entire range (70–90 vol%) were observed, which led to poor conversions at 24 h (19–37%) (Table 1, entries 1–3). Activity losses were also observed in ChCl–EG, yet an order of magnitude higher than for ChAc–Gly, which led to conversions >99%–73% after 24 h (Table 1, entries 4–6). Activities improved slightly for ChCl–Gly, but still remained low compared to pure buffer (50 mM KPi, pH 6), leading to moderate conversions (Table 1, entries 7–8). Remarkably, the enzymatic activity in 70 vol% Bet–Gly (13.0 ± 1 U·mg^−1^) was higher than that in buffer (12.0 ± 0.2 U·mg^−1^, Table 1, entries 10 and 13). The activity drops slightly when Bet–Gly proportions of 80–90 vol% were used, yet values remained high compared to the other DES systems (**Table** 1, entries 11‐12). Excellent conversions were achieved in the 70–80 vol% systems, while a significant decrease was observed for DESs at 90 vol% (Table 1, entries 10–12). Replacing choline chloride with betaine in DES used for biocatalysis is gaining importance, as halide‐free solvents are aligned with better enzymatic activities, as shown herein for PAD, or elsewhere for oxidoreductases.^[^ ^64^, ^66^ ^]^

**Table 1:** FA conversion at 10 mM (1.9 g L^−1^) after 24 h at 30 °C in different DES–water systems in 1 mL with 100 μg CFE of PAD N31. Buffer (50 mM KPi, pH 6) represents the water added to prepare the DES–water formulations. Reactions were performed as biological duplicates.

Entry	DES (1:2 mol:mol)	DES content/vol%	Activity/U·mg^−1^	FA Conversion/%
1	ChAc–Gly	70	0.015 ± 0.004	37.0 ± 7.0
2		80	0.014 ± 0.01	28.0 ± 18.0
3		90	0.005 ± 0.002	19.4 ± 0.9
4	ChCl–EG	70	0.9 ± 0.2	99.3 ± 0.5
5		80	0.7 ± 0.3	96.0 ± 2.0
6		90	0.12 ± 0.06	73.0 ± 3.0
7	ChCl–Gly	70	1.60 ± 0.04	83.0 ± 2.0
8		80	1.6 ± 0.3	83.0 ± 2.0
9		90	1.2 ± 1.0	12.0 ± 3.0
10	Bet–Gly	70	13.0 ± 1.0	97.9 ± 0.1
11		80	9.4 ± 0.2	96.3 ± 0.1
12		90	7.0 ± 2.0	47.0 ± 6.0
13	Buffer	–	12.0 ± 0.2	100.0 ± 0.0

To gain further insights into PAD N31, Michaelis–Menten kinetics parameters were determined in three DESs (80 vol%), excluding ChAc–Gly due to the poor performance in previous studies. The *K*_M_,_FA_ and *V*_max_ values showed high standard deviations, attributed to the relatively high viscosity of the DES systems (Figure S5 and Table S1, Supporting Information). The enzyme kinetics analyses indicate that the *K*_M_ value generally increases with the addition of DES, particularly affecting solvation of the substrate at the enzyme's active site. For ChCl–EG, separate *K*_M_ and *V*_max_ values could not be determined, suggesting a very high *K*_M_.^[^ ^61^ ^]^ Using first‐order kinetics of Michaelis–Menten kinetics, *V*_max_/*K*_M_ was calculated at 0.19 ± 0.008 U·mg^−1^·mM^−1^. ChCl–Gly shows strong substrate inhibition with a K_i_ of 0.3 ± 2.6 mM, while *K*_M_ and *V*_max_ have high standard deviations (126 ± 1222 mM and 236 ± 2307 U·mg^−1^, respectively), yielding a *k*_cat_ of 5.6 ± 10 1/s. Bet–Gly shows no substrate inhibition, and *K*_M_ and *V*_max_ values are 9 ± 2 mM and 4.6 ± 0.4 U·mg^−1^ (Table S1, Figure S5, Supporting Information) with a *k*_cat_ of 1.6 ± 0.2 1/s. Overall, the activity and kinetic screening revealed Bet–Gly (1:2) at 80 vol% as the prime candidate for further studies, as it displayed the best activity (9.4 ± 0.2 U·mg^−1^) and conversion (96.3 ± 0.1%) (Table 1, entry 11) with no substrate inhibition at higher substrate loading during kinetic screening.

#### 2.2.2 Stability of PAD N31

**Figure 3:**
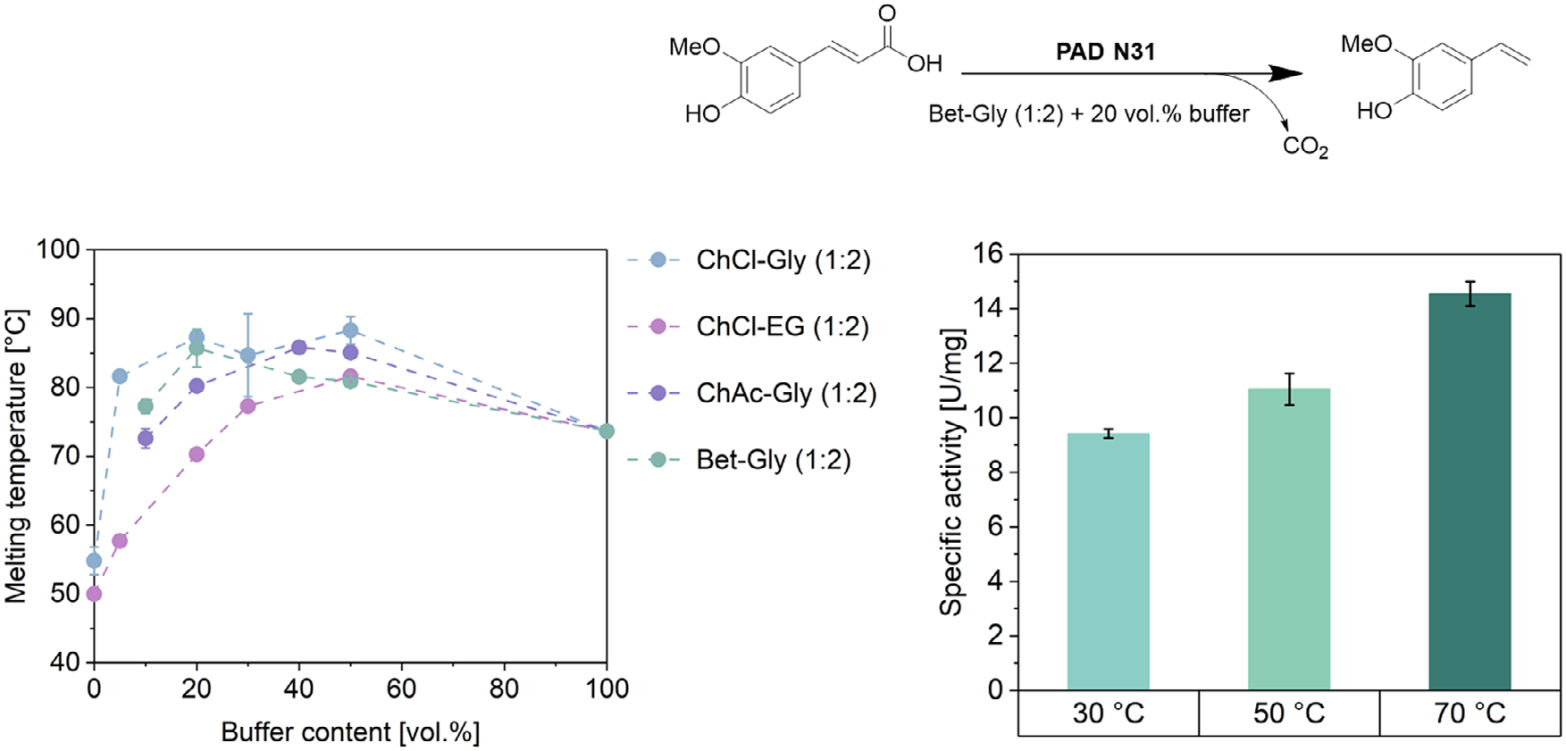
Left: Melting temperature (*T*_m_) of PAD N31 in various DES–buffer mixtures containing 0–100 vol% buffer (50 mM, KPi buffer, pH 6.0). Lines connecting experimental data points are provided for illustration purposes only. Right: Specific activity in 80 vol% Bet–Gly (1:2) at three different temperatures (30, 50, and 70 °C). Experiments were performed in triplicate.

The enzyme stability in the DESs was evaluated by determining the melting temperature (*T*_m_) using NanoDSF (NanoTemper Technologies GmbH, Germany) and validated by measuring the half‐life times. The *T*_m_ values were measured in DES–buffer mixtures with buffer content ranging from 0 to 100 vol%. The presence of DES can enhance the stability in a range of 20–50 vol% buffer. PAD N31 shows a high melting temperature of 75 °C in buffer compared to the wild‐type *Bs*PAD (54 °C). ChCl–EG (1:2) showed detrimental effects on the enzyme by the addition of 50 vol% DES (**Figure** **3**, light pink points). The stabilizing effects of DESs, particularly those based on glycerol, have been studied for several enzymes and are attributed to a solvation layer around the enzyme, as described for HLADH in detail through molecular dynamics simulations. In our studies, we observed that ChAc–Gly (1:2) has unfavorable effects on the enzyme after 40% vol. of buffer (Figure 3, light violet points), whereas it stabilizes the enzyme in a range of 100–40 vol% buffer with the highest *T*_m_ at 85.8 ± 0.2 °C. PAD N31 displays stabilizing effects in ChCl–Gly in the range of 50–20 vol% buffer (Figure 3, light blue points). The melting temperature increased by 38% compared to the pure buffer. Afterwards, negative effects are observed, following the measured activity at different DES contents. Adverse effects on enzyme activity were also observed above 80 vol% DES. A similar trend is detected for Bet–Gly (Figure 3, light green points). Hereby, the highest *T*_m_ was observed with 20 vol% buffer (=80 vol% DES) at 85.7 ± 3.0 °C.

Next to the PAD N31's melting temperature, also its half‐life time in different DES‐based media was analyzed (Figure S7, Supporting Information). PAD N31 shows decreased half‐life time in 80 vol% ChCl–EG (1:2) with 1.5 ± 0.2 h, whereas the stability increases drastically in ChCl–Gly and Bet–Gly. No half‐life time could be determined for 80 vol% ChAc–Gly (1:2) since no product formation was detected after 24 h of reaction time.

An evaluation of enzyme stability shows that ChAc–Gly and ChCl–EG perform worse compared to ChCl–Gly and Bet–Gly, while the latter exhibit comparable performance, with a *T*_m_ value of 87.3 ± 0.9 °C and 85.7 ± 3.0 °C, respectively, and half‐life times exceeding 15 days. This observation can be explained by the combined effects of the DES components: The stabilizing influence of glycerol outweighs the destabilizing effect of HBA choline acetate (ChAc) or choline chloride (ChCl). Notably, in the particular case of PADs, the denaturing effect on the enzyme of ChAc is considerably more pronounced than that of ChCl. Whereas a beneficial influence of betaine on enzyme stability was observed. Overall, Bet–Gly DESs, albeit displaying lower solubility, appear to be a suitable candidate for further intensification measures.

### 2.3 From Millimolar to Molar Scale. Process Intensification with Thermostable PAD N31 in Bet–Gly DES (80 vol%)

When all aspects assessed in previous sections, i.e., solubility of FA, viscosity of DES–water formulations, the influence of buffer in the system, and the right temperature to apply, are combined, 80 vol% Bet–Gly (1:2) results in a promising candidate, even though only moderate FA solubility (50 mM) was observed. This solvent surpassed the other DESs in terms of the enzyme activity (9.4 ± 0.2 U·mg^−1^) and subsequently achieved excellent conversions (96.3 ± 0.1%) under the analyzed conditions. The reason why 100% conversion could not be observed can be attributed to the partial agglomeration of the enzyme that was noted. Attempting sequential enzyme addition did not achieve the desired outcome, as the reaction medium is already very heterogeneous at this stage, causing the enzyme to form agglomerates.

Furthermore, high enzyme stability was observed with a *T*_m_ value of 86 ± 3 °C and a half‐life time greater than 15 days. Finally, designing halide‐free DESs may contribute to milder waste treatments in their fate. To our knowledge, PAD enzymes have never been assessed in Bet‐based DESs, nor at molar substrate concentrations. With the selected Bet–Gly‐water (80 vol%) in hand, the reaction temperature was increased, taking advantage of a thermostable enzyme. As stated above, a higher temperature leads to better substrate solubility, lower medium viscosity, and faster reaction rates. Reactions were performed at 30–70 °C, showing that the enzyme deactivated at 70 °C within 24 h, whereas it remained stable at 50 °C.

**Figure 4:**
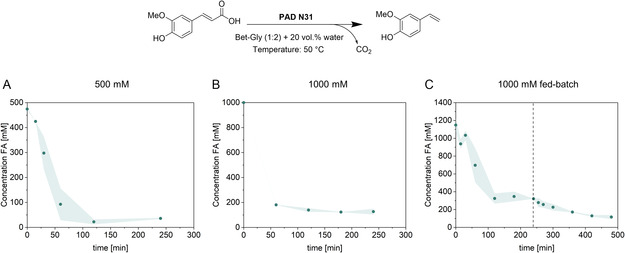
A) 500 mM ferulic acid in 80 vol% Bet‐Gly (1:2) with 50 mg PAD N31 CFE (388 U) at 50 °C, 640 rpm, and 1 mL reaction volume. B) 1000 mM ferulic acid in 80 vol% Bet–Gly (1:2) with 90 mg PAD N31 CFE (698 U) in 80 vol% Bet–Gly (1:2) at 50 °C, 640 rpm, and 1 mL reaction volume. C) Fed‐batch reaction with 1000 mM ferulic acid in 80 vol% Bet–Gly (1:2) with 45 mg CFE (349 U) at 0 h and 4 h at 50 °C, 640 rpm, and 1 mL reaction volume.

The reaction was intensified to 500 mM FA (≈97 g L^−1^) under slurry reaction conditions (**Figure** **4**, left; Figure S8, Supporting Information), following an analogous procedure as reported by Schweiger et al.^[^ ^51^ ^]^ Gratifyingly, a conversion of >90% was achieved within four hours. The substrate concentration was then further increased to 1 M (≈194 g L^−1^), leading also to excellent conversions. With the addition of more substrate to 1.5 and 2 M, the reaction slurry solidified. Thus, 1 M was selected for further investigations (Figure 4, middle). To achieve complete FA conversion within 8 h, a fed‐batch strategy was applied. Therefore, two batches of enzyme were added at the beginning and after 4 h, leading to a conversion of 88% at 8 h. Increasing the enzyme loading, or adding it in more portions, did not lead to higher conversion rates.

Therefore, the substrate titer was increased by a factor of 2.5 (400 vs. 1000 mM) compared to previous studies of the same reaction, conducted either in other organic solvents or in ChCl–Gly DESs (**Table** **2**). The identification of new processing conditions has been possible by fine‐tuning the DES nature, its water content, and the reaction temperature by incorporating thermostable enzymes. A suitable Bet–Gly DES enhances enzyme stability while maintaining high activity, even at elevated DES concentrations, thus increasing the volumetric productivity to 17 g·L^−1^·h^−1^ at considerably high substrate loadings (≈200 g L^−1^) (Table 2). While CPME shows promising performance as an organic solvent, DES‐based systems offer a key advantage for thermostable enzymes, enabling higher reaction temperatures (50–70 °C) without solvent loss and keeping enzyme activity. The observed difference in productivity (CPME vs. DESs) may be tackled in future works through protein engineering campaigns to tailor new PADs with more adapted performance to the proposed DESs.

**Table 2:** Comparison of the PAD‐catalyzed decarboxylations in the respective reaction media. *Bs*PAD: wild‐type PAD from *Bacillus subtilis*.

PAD	Reaction medium	*c*(Sub.)	Conversion %, time	Scale	Volumetric productivity [g_product_·L^−1^·h^−1^]	Biocatalyst productivity [g_product_·g_enzyme_^−1^·h^−1^]	Reference
*Bs*PAD	wet CPME	0.63 M FA (≈122 g L^−1^)	99%, 4.5 h	10 L	27	2.6	[32]
*Bs*PAD	ChCl–Gly, 50 vol%	0.4 M CA (≈72 g L^−1^)	77%, 24 h	1 mL	2	0.07	[51]
PAD N31	ChCl–Gly, 70 vol%	0.4 M FA (≈78 g L^−1^)	70%, 24 h	1 mL	1.8	0.18	[22]
PAD N31	Bet–Gly, 80 vol%	1 M FA (≈200 g L^−1^)	90%, 8 h	1 mL	17	0.19	this study

Another important aspect in process design is the downstream processing of the produced compounds to achieve on‐spec, marketable conditions. While downstream units with classic organic solvents typically involve distillation procedures with high efficiency, the use of DESs (and other neoteric solvents) poses new challenges that need to be tackled, considering the recyclability of the solvent and its potential degradation over reaction loops. Herein, liquid–liquid separations were preliminarily assessed in the lab by using (potentially biogenic) ethyl acetate as the extractive agent. After filtration of the (unreacted) ferulic acid, the pH of the DESs containing 4‐VG and FA was adjusted to 10 by adding carbonate buffer, which led to deprotonation of the remaining FA in the DES. Subsequently, 4‐VG was selectively extracted with ethyl acetate (3X), dried, and then distilled under reduced pressure. By applying that protocol, no DES components were extracted, whereas the product, 4‐VG, could be separated with a yield of up to 15% and high purity. While the nonoptimized procedure clearly shows room for improvement, establishing liquid–liquid separation devices may be beneficial when working with DES‐based media and thermostable enzymes. An important environmental and economic aspect herein would be the setup of an adequate solvent recycling system to ensure that the environmental burden associated with classic solvents remains as low as possible.^[^ ^67^ ^]^ Research devoted to integrating DESs and downstream processing procedures is becoming timely to reach a holistic processing design with adequate sustainability. Importantly, as observed in our experiments, the DES components remained stable throughout the entire operability of the reaction, with no signs of degradation noted.

## 3 Conclusion

DESs hold potential for intensifying phenolic acid decarboxylation with ancestor PAD N31 to valorize lignin‐derived raw materials. Several DESs were assessed based on substrate solubility, water content, temperature, and enzymatic parameters (activity, kinetics, stability), to select the best‐designed candidate as a reaction medium. 80 vol% Bet–Gly (1:2) exert beneficial properties for the reaction, such as: i) Moderate substrate solubility (9.7 ± 0.7 g L^−1^), which can be enhanced by working under a slurry‐type system; ii) high enzyme stability, which was confirmed with the melting temperature (*T*_m_ of 81.5 °C) and half‐life time (no activity loss after 15 days); iii) high enzymatic activity (9.4 ± 0.2 U·mg^−1^); and iv) incorporation of halide‐free media that can contribute to less harsh waste treatments. On the basis of the results, the decarboxylation was successfully applied to industrially relevant substrate loadings (≈200 g L^−1^) using a fed‐batch process at elevated temperatures (50 °C), leading to excellent conversions and productivities in short reaction times (up to 8 h). The findings untap the potential for enzymatic decarboxylations to be applied on an industrial scale with relevant substrate concentrations and volumetric productivities (>10 g_product_·L^−1^·h^−1^), also for bulk chemical production.^[^ ^68^ ^]^ This process improvement was accompanied by an increased loading and yield, which, in turn, reduces waste generation (less volume of media to be used for production).

For future considerations, to realize full conversion in the reaction, several obstacles need to be addressed. One critical factor is preventing enzyme agglomeration; therefore, it is crucial to eliminate the denatured and precipitated enzyme prior to introducing new enzyme into the system, thereby avoiding solution heterogenization. Moreover, the use of a fed‐batch strategy for the incremental addition of the substrate could prove to be a promising method that demands further investigation. Additionally, the process needs to be scaled up to relevant levels, and considerations related to downstream must be addressed (e.g., by establishing liquid–liquid separations or precipitation units). Additionally, enzyme immobilization may be an effective strategy to enhance enzyme stability, facilitating the reuse of the enzyme for multiple cycles and thereby improving cost and waste efficiency.^[^ ^4^, ^69^ ^]^ To further minimize waste generation and reduce overall process costs, the reuse of DESs should be considered. Integrating DES recovery into the downstream processing not only complements economic objectives but also significantly contributes to the sustainability evaluation of the overall process. Future research will focus on these lines to further demonstrate that DES media can be enzyme‐compatible and useful for practical synthetic procedures.

## Conflict of Interest

The authors declare no conflict of interest.

## Supporting information

Supplementary Material

## Data Availability

The data that support the findings of this study are available in the supplementary material of this article.
