# Supplementary material for: Molar‐Scale Phenolic Acid Decarboxylation Using Thermostable Biocatalysts and Enzyme‐Compatible Deep Eutectic Solvents
Source: ChemSusChem. 2025 Nov 9;18(24):e202501755. doi: 10.1002/cssc.202501755 (PMC12703459; doi:10.1002/cssc.202501755)
Supplement: Supplementary file 1 — Supplementary Material [file CSSC-18-e202501755-s001.pdf]

Supporting Information

Molar-scale phenolic acid decarboxylation using thermostable biocatalysts and enzyme-compatible deep eutectic solvents

Sonja Vaupel[a], Lars-Erik Meyer[a], Pablo Domínguez de María[b] and Selin Kara*[a],[c]

[a] M.Sc., S., Vaupel; Dr., L.-E., Meyer; Prof. Dr.-Ing. habil., S., Kara
Institute of Technical Chemistry
Leibniz University Hannover
Callinstr. 3, 30167 Hannover, Germany
E-mail: selin.kara@iftc.uni-hannover.de

[b] Dr. habil., P. Domínguez de María
Sustainable Momentum, SL.
Av. Ansite 3, 4-6, 35011, Las Palmas de Gran Canaria, Canary Islands, Spain

[c] Prof. Dr.-Ing. habil., S., Kara
Biocatalysis and Bioprocessing Group
Department of Biological and Chemical Engineering
Aarhus University
Gustav Wieds Vej 10, 8000 Aarhus C, Denmark
E-mail: selin.kara@bce.au.dk

1 Table of contents

1. MATERIAL AND METHODS 3
1.1 Preparation of lyophilized PAD N31 3
1.2 High-performance liquid chromatography 3
1.3 Characterization of DES and DES-buffer mixtures 4
1.3.1 Preparation of DESs and DES–water mixtures 4
1.3.2 Substrate solubility evaluation 4
1.3.3 Determination of the viscosity 4
1.4 Characterization of the enzymatic DES reaction system 4
1.4.1 Activity assay in aqueous media 4
1.4.2 Protein assay 5
1.4.3 Conversion of FA in DESs 5
1.4.4 Michaelis-Menten kinetics in buffer 5
1.4.5 Michaelis-Menten kinetics in DESs 5
1.4.6 Enzyme stability 5
1.4.7 Half-life time analysis 6
1.5 Intensified biocatalytic decarboxylation 6
2 ADDITIONAL INFORMATION 7
2.1 SDS-PAGE analysis of the CFE of PAD N31 7
2.2 Exemplary HPLC chromatograms and calibration of ferulic acid in different DESs 8
2.3 Activity of PAD N31 in DES with different water contents 10
2.4 Specific activity of PAD N31 towards FA with various concentrations in DES 11
2.5 NanoDSF measurement for determining the stability of PAD N31 in DES 12
2.6 Half-life time of PAD N31 13
2.7 Reaction of 500 mM ferulic acid in 80 vol.% Bet-Gly 13
2.8 GC-MS results 14

1. Material and Methods

All chemicals, materials and solvents were obtained from commercial suppliers (Acros Organics, Alfa Aesar, Sigma-Aldrich, VWR International, Carl-Roth GmbH, Merck KGaA, Thermo Fisher Scientific, TCI Europe, Biowest) and used as received: *trans*-ferulic acid (FA, ≥99% grade), K_2HPO_4 (≥99%), KH_2PO_4 (≥99%), NaOH (≥98%, p.a.), aqueous HCl (37%), dimethyl sulfoxide (DMSO, ≥99.5%), choline chloride (ChCl, 99%), glycerol (Gly, 99%), ethylene glycol (EG, ≥98.0%), betaine (Bet, ≥99.0%), choline acetate (ChAc, 98%), Karl-Fischer solvent CM (Art. No. 85461.290), Karl-Fischer reagent TitrANT 5 (Art. No. 85468.320), HYDRANAL™ Standard 5.0 (Art. No. 34813), Bradford assay solution (Art. No. B5702), bovine serum albumin (BSA, 2 mg/mL standard), formic acid (≥95%). Acetonitrile (≥99.95%, UHPLC grade) used for HPLC was purchased from VWR International.

1.1 Preparation of lyophilized PAD N31

The heterologous expression of phenolic acid decarboxylase (PAD) N31 was performed in E. coli BL21 (DE3) containing the plasmid of pET-28b (+)-PAD N31-His.The plasmid contains the PAD N31 gene with an N-terminal His-tag and kanamycin resistance. The plasmid was kindly provided by Prof. Dr. Robert Kourist and Dr. Daniel Kracher (Graz University of Technology, Austria). The preculture was grown in 20 mL of LB medium containing 50 µg mL^{-1} kanamycin, which was incubated at 37°C and 100 rpm for 16 h. A small fraction of the preculture was used to prepare a glycerol stock with 20 vol.% glycerol to be stored at -80°C. Meanwhile, 10 mL of the preculture (1 vol.%) was used to inoculate 1 L LB medium containing 50 µg mL^{-1} kanamycin. The main culture was incubated at 37°C and 80 rpm until an OD_{600} of 1.5−3.0 was reached. Subsequently, the enzyme expression was induced by adding IPTG to have a final concentration of 1 mM. The incubation was continued at 20°C for 21 h. To harvest the cells, the culture was centrifuged at 4700 rpm for 10 min. To generate the cell-free extract (CFE), the cell pellets were resuspended in a ratio of 1 g wet cells per 6 mL ddH_2O and ultrasonically disrupted on ice (65% amplitude, 0.2 cycles, 2 sec on, 8 sec off, 2 min, 6 cycles). The water-soluble protein was separated from the cell debris by centrifugation at 13,000 rpm and 4°C for 45 min, called cell-free extract (CFE). The obtained CFE was filtered through a 0.2 µm sterile filter and then lyophilized.

1.2 High-performance liquid chromatography

Both qualitative and quantitative analyses were performed with a Hitachi Chromaster high-performance liquid chromatography system (Japan) consisting of a 5160 quaternary pump, a 5260 standard auto sampler, a 5310 column oven, and a 5430 DAD detector. A Kinetex® 2.6 µm F5 Core-Shell LC column (50 × 4.6 mm, Phenomenex) was used. The sample injection volume was 10 µL, the column oven temperature 30 ± 0.5 °C, and the total flow rate 0.8 mL min^{-1}. A gradient HPLC method of 0.1% formic acid in water (A) and acetonitrile (B) was used. The initial mobile phase composition was 95% A and 5% B, then ramped up to 20%

A and 80% B over 5 min and then held constant for 2 min. Afterwards, the gradient declined within 1 min to the initial mobile phase composition of 95% A and 5% B and held stable for further 7 min. The 310 nm channel of the diode array detector (DAD) was used for calibration and quantification of ferulic acid.

1.3 Characterization of DES and DES-buffer mixtures

1.3.1 Preparation of DESs and DES–water mixtures

For ChCl-Gly (1 : 2), choline chloride and glycerol were weighed in a flask in a molar ratio of 1 : 2 [0.25 mol (34.9 g) of ChCl : 0.5 mol (46.0 g) Gly]. The mixture was heated and stirred at 60 °C and 300 rpm until a colorless liquid was formed (*ca.* 1 hour).

For ChCl-EG (1 : 2), choline chloride and ethylene glycol (EG) were directly weighed in a flask in a molar ratio of 1 : 2 [0.25 mol (34.9 g) of ChCl: 0.5 mol (31.0 g) of EG]. The mixture was heated and stirred at 60 °C and 300 rpm until a colorless liquid was formed (*ca.* 2 hours).

For Bet-Gly (1 : 2), betaine (Bet) and glycerol were directly weighed in a flask in a molar ratio of 1 : 2 [0.25 mol (29.3 g) of Bet: 0.5 mol (31.0 g) of Gly]. The mixture was heated and stirred at 80 °C and 300 rpm until a colorless liquid was formed (*ca.* 2 hours).

All DESs–water ratios are given in vol.% with DESs and KPi (50 mM, pH 6).

1.3.2 Substrate solubility evaluation

Substrate was added in excess, and the suspensions were stirred for 2–5 h at 300 rpm at 30 °C. Afterwards, the mixtures were transferred to 1.5 mL microcentrifuge tubes, centrifuged (13,400 rpm, 2 min), and subsequently, the supernatants were sampled and diluted in a series of dilutions (10×, 100×, 500×, 1000×) using water/acetonitrile (1/1, v/v). These dilutions were subjected to HPLC-UV analytics. From peaks within calibration range, solubilities were derived in mmol L^{-1}.

1.3.3 Determination of the viscosity

The viscosity of DES and DES-water mixtures was determined by rheometric measurements with a Modular Compact Rheometer MCR302 by Anton Paar (Germany) with a PP40 measuring plate. Therein, 750 µL samples were applied to the device, and the viscosity was measured for increasing shear rates for 1–1000 1/s at temperatures of 30 °C to 70 °C. In cases where the viscosity value stabilized as shear rates increased, the viscosity was defined as the average of the last 5 data values.

1.4 Characterization of the enzymatic DES reaction system

1.4.1 Activity assay in aqueous media

This standard assay was performed in KPi buffer (50 mM, pH 6.0) using ferulic acid as substrate at an initial concentration of 10 mM, ensuring zero-order kinetics. For the former, 950 µL KPi buffer were mixed with 50 µL FA stock (200 mM in DMSO) and heated to +30 °C.

To start the reaction, 10 µg purified enzyme was added and the mixture was subjected to +30 °C and 1200 rpm. Samples of each 40 µL were quenched and diluted by addition to 960 µL water/acetonitrile (1/1, v/v) and subjected to HPLC analysis. Standard sampling times were 0, 2, 3, 4 and 5 min. All derived kinetic data is based on substrate depletion.

1.4.2 Protein assay

For the determination of the protein content, the Pierce^{TM} BCA Protein Assay Kit was used according to the manufacturer's instruction.

1.4.3 Conversion of FA in DESs

The determination of the conversion of FA within 24 h in DESs was investigated with 30 vol.%, 20 vol.% and 10 vol.% of KPi (50 mM, pH 6.0). A stock solution of 60 mM FA in DES was prepared (e.g. 116.5 mg in 10 mL in DES at room temperature). The reaction mixture consisting of 80 vol.% DES with 10 mM FA was prepared and 136 µg purified enzyme was added to reach a final volume of 1 mL. Samples of 40 µL were withdrawn, quenched and diluted by the addition of 960 µL water/acetonitrile (1/1) and subjected to HPLC analysis. Standard sampling was taken after 0, 30, 60, 120, 240 and 1440 min. All derived kinetic data are based on substrate depletion.

1.4.4 Michaelis-Menten kinetics in buffer

Michaelis-Menten kinetics were performed in KPi (pH 6, 50 mM). Therefore, 50 µg CFE was added to 0 – 4 mM FA (from a 200 mM DMSO stock) in KPi (50 mM, pH 6.0) at a final volume of 1 mL. Samples of 40 µL were quenched and diluted by the addition to 960 µL water/acetonitrile (1/1 v/v) and subjected to HPLC analysis. Standard sampling times were 0, 2, 3, 4 and 5 min. All derived kinetic data is based on substrate depletion.

1.4.5 Michaelis-Menten kinetics in DESs

Michaelis-Menten kinetics were performed in 80 vol.% DESs. Therefore, 103 µg purified enzyme was added to 0 – 30 mM FA (from a 200 mM DMSO stock) in different DESs. Samples of 40 µL were quenched and diluted by the addition of 960 µL water/acetonitrile (1/1 v/v) and subjected to HPLC analysis. Standard sampling was taken at 0, 2, 3, 4 and 5 min. All derived kinetic data is based on substrate depletion.

1.4.6 Enzyme stability

The melting temperature of enzymes was recorded with NanoDSF (Nano Temper Technologies GmbH, Germany). The purified enzyme solution (1 mg/mL) in KPi buffer (50 mM, pH 6.0) was loaded in capillaries. The fluorescence change was recorded over the 20–95°C temperature range at a ramp rate of 1°C/min.

1.4.7 Half-life time analysis

Aliquots of 60 µl of a defined amount (0.2 mg/mL) of the enzyme were incubated at 60°C in a DES-buffer mixture consisting of 20 vol.% KPi buffer (pH 6, 50 mM) and 80 vol.% DES (ChCl-Gly, Bet-Gly, and ChCl-EG (1:2)). The residual activities were measured with the standard activity assay in buffer by HPLC. Half-life time was determined from the plots of the natural logarithm of residual activities versus the incubation time. All measurements were performed in duplicates.

1.5 Intensified biocatalytic decarboxylation

Ferulic acid (1 M) was suspended in 80 vol.% Bet-Gly (1:2) in a final volume of 1 mL. To start the reaction, 90 mg CFE was added, and the reaction mixture was put on a thermo shaker at 50°C and 540 rpm. Samples of 10 µL were withdrawn after 0, 60, 120, 180, 240, and 1440 min and quenched with 990 µL acetonitrile/water (1/1). Afterwards, the samples were vortexed, centrifuged (13,200 rpm, 2 min), and further diluted for HPLC analysis.

2 Additional Information

2.1 SDS-PAGE analysis of the CFE of PAD N31

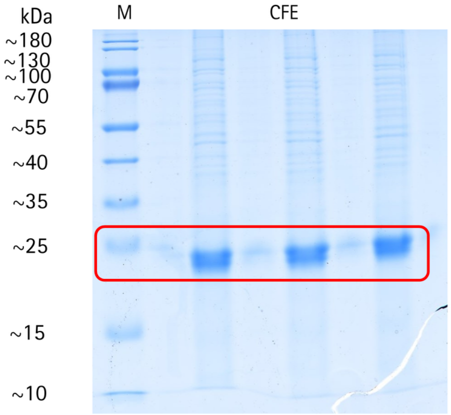

Figure S1. SDS-PAGE (12%) analysis of the CFE of PAD N31. CFE: cell-free extract, M = PageRuler Prestained Protein Ladder (Thermo Scientific). The M.W. of PAD N31 monomer is around 21 kDa (highlighted in red box).

2.2 Exemplary HPLC chromatograms and calibration of ferulic acid in different DESs

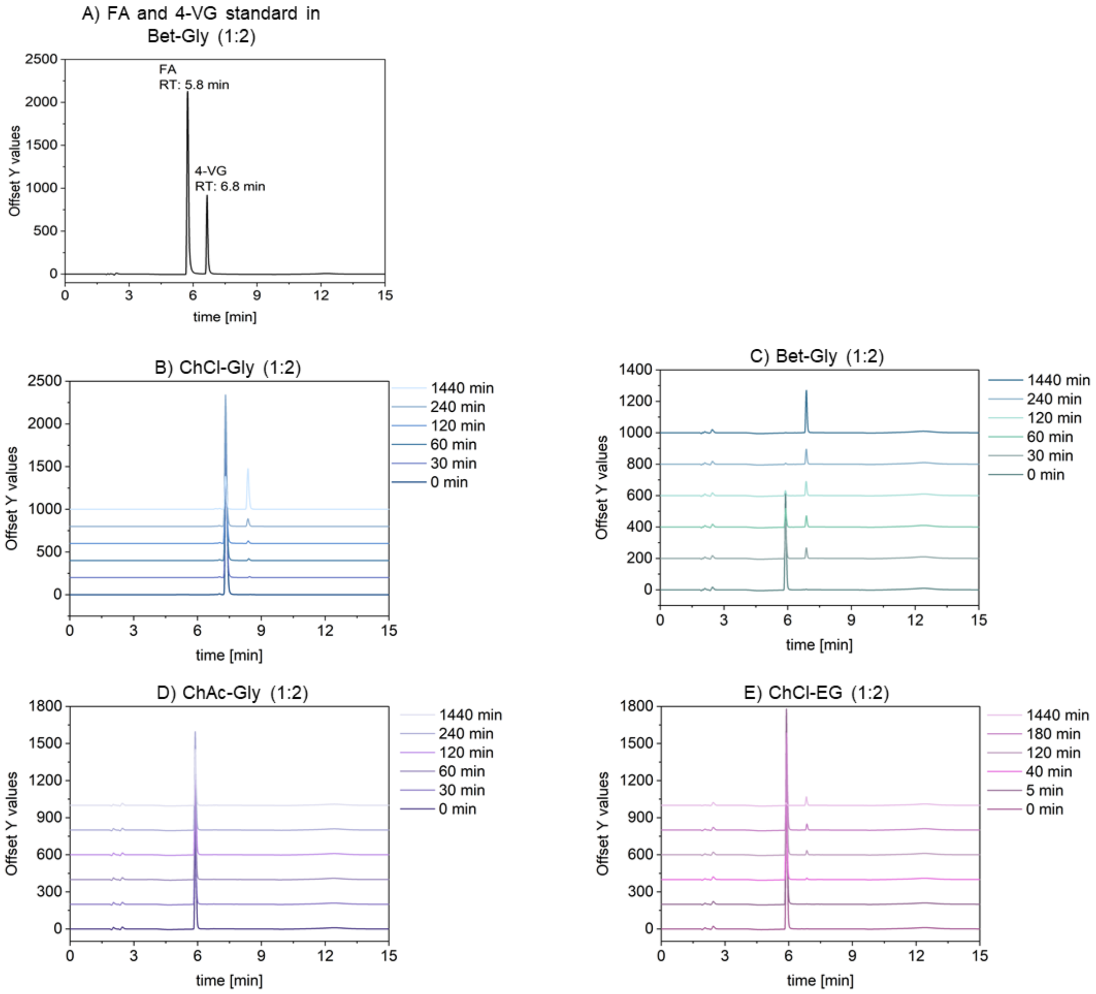

Figure S2. HPLC chromatograms of the reaction components FA (RT: 5.8 min) and 4-VG (RT: 6.8 min) in different DES-buffer mixtures. A) displays the components as reference standards in 80 vol.% Bet-Gly.

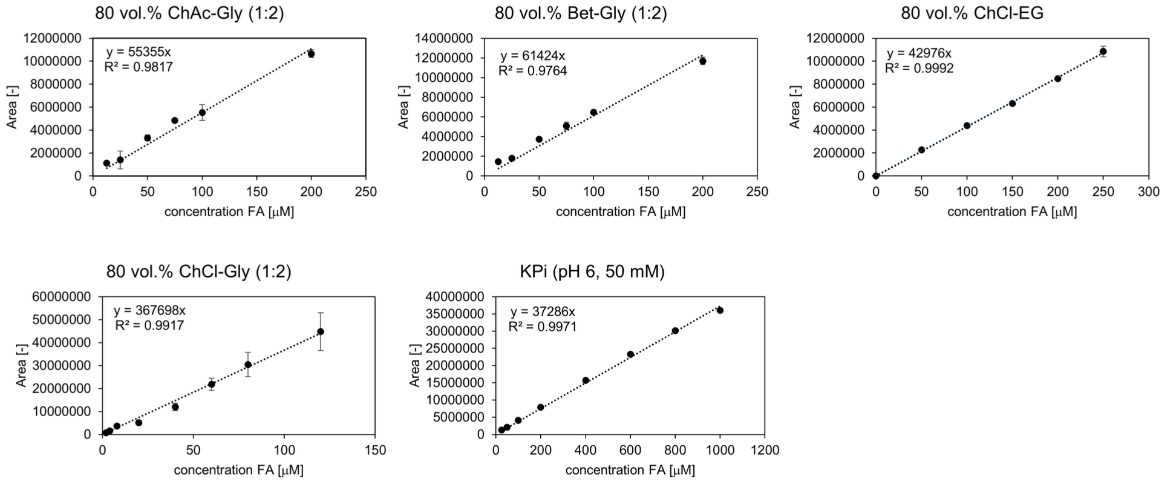

Figure S3. Calibration of ferulic acid in different DESs. Triplicates in water/acetonitrile 1/1; 10 µL injection volume; $\lambda_{detection}$ = 310 nm.

2.3 Activity of PAD N31 in DES with different water contents

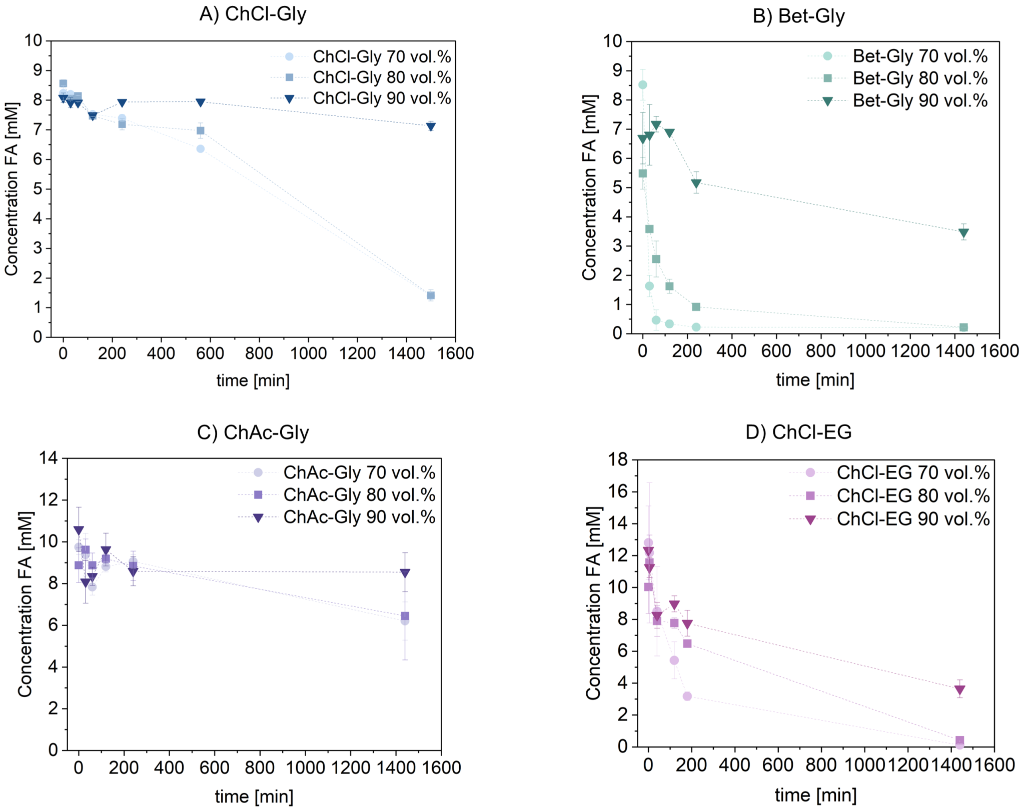

Figure S4. Activity of PAD N31 in DES with different water contents. The determination of activity was carried out in 1 mL of DES with 10 vol.%, 20 vol.%, and 30 vol.% buffer and 10 mM FA, using 10 µg of enzyme. The assay was performed at 30°C and 1200 rpm in a thermoshaker. Samples of 40 µL were taken after 0, 2, 3, 4, and 5 min and quenched with 960 µL water/acetonitrile (1/1). The samples were subjected to HPLC-UV analysis. A) ChCl-Gly (1:2), B) ChCl-EG (1:2), C) ChAc-Gly (1:2), and D) Bet-Gly (1:2).

2.4 Specific activity of PAD N31 towards FA with various concentrations in DES

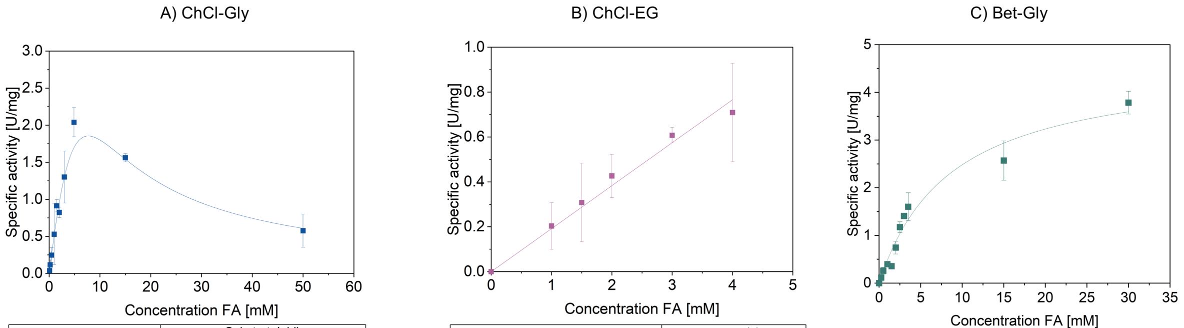

Modell	SubstrateInhib
Gleichung	y = Vmax * x / (Km + x * (1 + x / Ki));
Zeichnen	spec. acitivty
Vmax	15.77912 ± 26.36849
Km	28.94444 ± 51.66851
Ki	2.05237 ± 3.78842
Chi-Quadr Reduziert	0.00784
R-Quadrat (COD)	0.97853
Kor. R-Quadrat	0.97137

Gleichung	y = a + b*x
Zeichnen	activity
Gewichtung	Keine Gewichtung
Schnittpunkt mit der Y-Achse	0 ± --
Steigung	0.19153 ± 0.00649
Summe der Fehlerquadrate	0.00679
Pearson R	0.99714
R-Quadrat (COD)	0.9943
Kor. R-Quadrat	0.99316

Modell	MichaelisMenten
Gleichung	y = Vmax * x / (Km + x)
Zeichnen	activity
Vmax	4.62785 ± 0.40709
Km	8.6384 ± 1.73792
Chi-Quadr Reduziert	0.04867
R-Quadrat (COD)	0.96822
Kor. R-Quadrat	0.96469

Figure S5. Specific activity of PAD N31 towards FA with various concentrations in DES. The HPLC assay was performed with a final volume of 1000 µL containing 103 µg CFE and 0 to 50 mM FA. The reaction was carried out at 30 °C and 1200 rpm. Samples of 40 µL were taken after 0, 5, 10, 30, and 60 min and quenched with 960 µL acetonitrile/ water (1/1) and injected into HPLC analysis.

Table S1. Kinetic data of PAD N31 in different DES systems. Experiments were performed at 30°C (see also Figure S5).

	K_M / (mM)	V_{max} / ($U \cdot mg^{-1}$)	V_{max}/K_M / ($U \cdot mg^{-1} \cdot mM^{-1}$)	K_i / (mM)	k_{cat} / 1/s
Buffer	0.10 ± 0.03	5.0 ± 0.04	-	-	1.75 ± 0.02
ChCl-Gly (1:2) 80 vol.%	29 ± 52	16 ± 27	-	2 ± 4	5.6 ± 10.0
ChCl-EG (1:2) 80 vol.%	-	-	0.19 ± 0.008	-	
Bet-Gly (1:2) 80 vol.%	9 ± 2	4.6 ± 0.4	-	-	1.6 ± 0.2

2.5 NanoDSF measurement for determining the stability of PAD N31 in DES

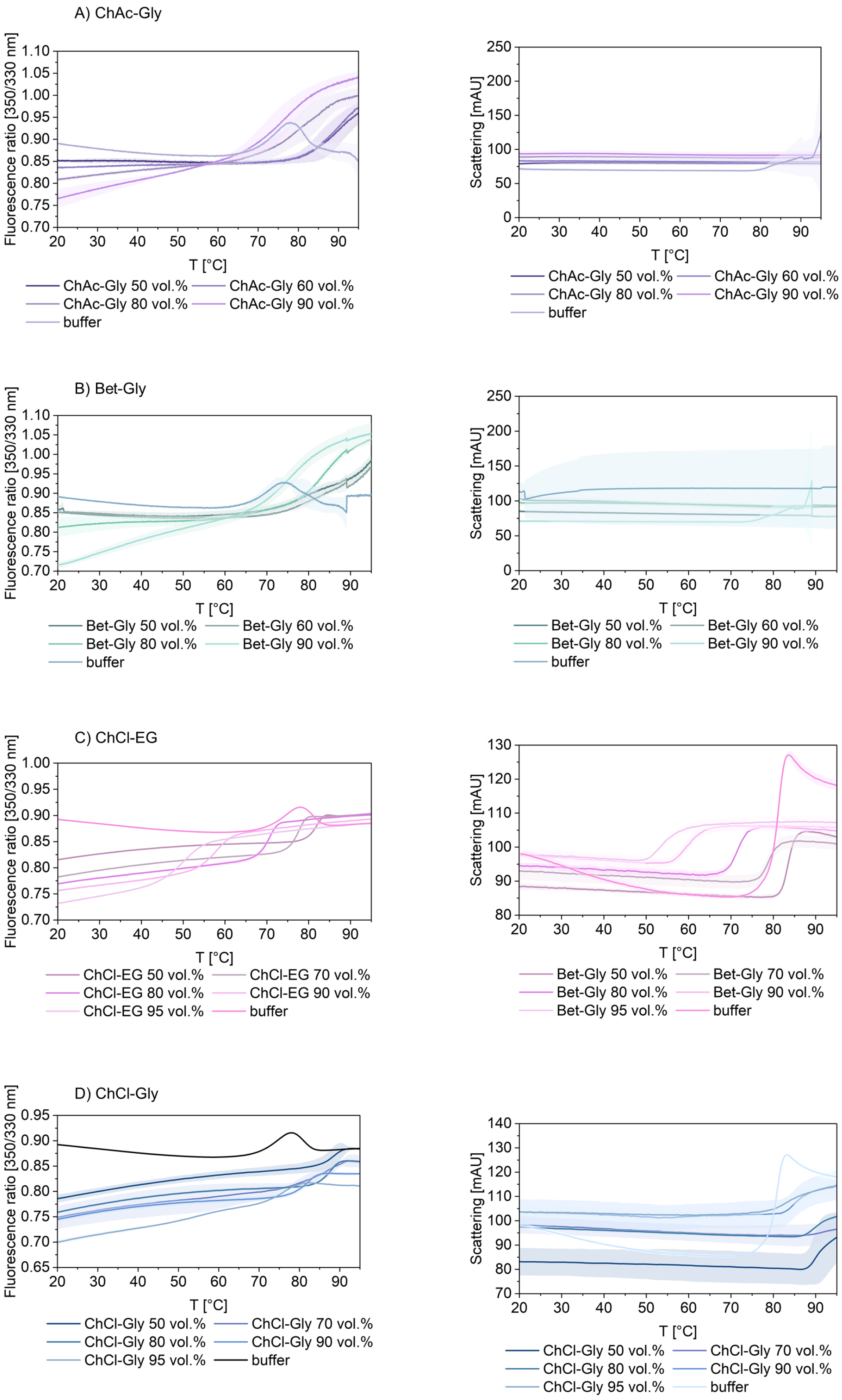

Figure S6. NanoDSF measurement for determining the T_m (left) and T_{agg} (right) of PAD N31 in DES with varying buffer contents: A) ChAc-Gly (1:2), B) Bet-Gly (1:2), C) ChCl-EG (1:2), D) ChCl-Gly (1:2). The error bars are based on triplicate experiments.

2.6 Half-life time of PAD N31

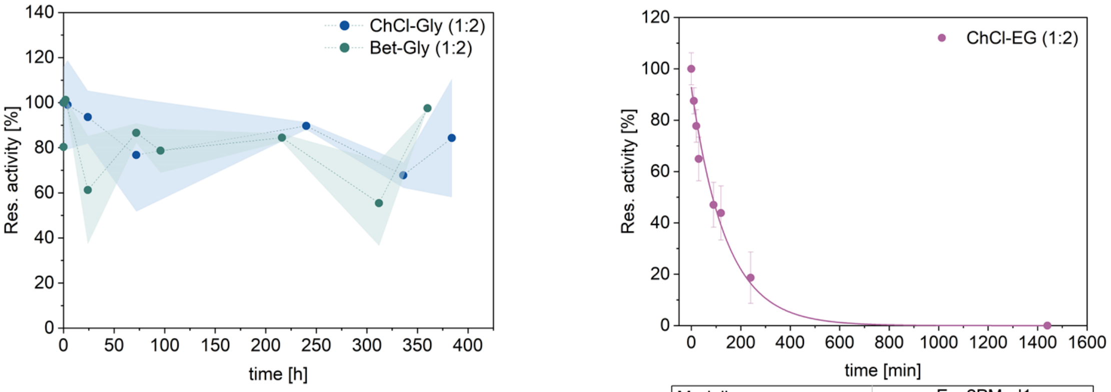

Modell	Exp2PMod1
Gleichung	y = a*exp(b*x)
Zeichnen	activity
a	92.73627 ± 3.52021
b	-0.00721 ± 8.46058E-4
Chi-Quadr Reduziert	31.11726
R-Quadrat (COD)	0.97733
Kor. R-Quadrat	0.97355

Figure S7. Half-life time of PaD N31. Purified PAD N31 was incubated in 80 vol.% DES in 60 µL aliquots at 60 °C (Right: ChCl-Gly, Bet-Gly; left: ChCl-EG). The determination of the activity was carried out in 1 mL KPi (50 mM, pH 6) and 10 mM FA with 10 µg enzyme. The assay was performed at 30°C and 1200 rpm in a thermoshaker. Samples of 40 µl were taken after 0, 2, 3, 4, and 5 min and quenched with 960 µl water/acetonitrile (1/1). The samples were subjected to HPLC-UV analysis.

2.7 Reaction of 500 mM ferulic acid in 80 vol.% Bet-Gly

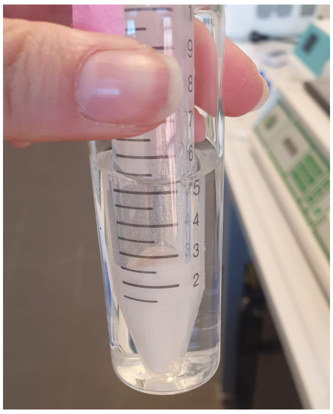

Figure S8. Reaction mixture of 500 mM FA before enzyme addition.

2.8 GC-MS results

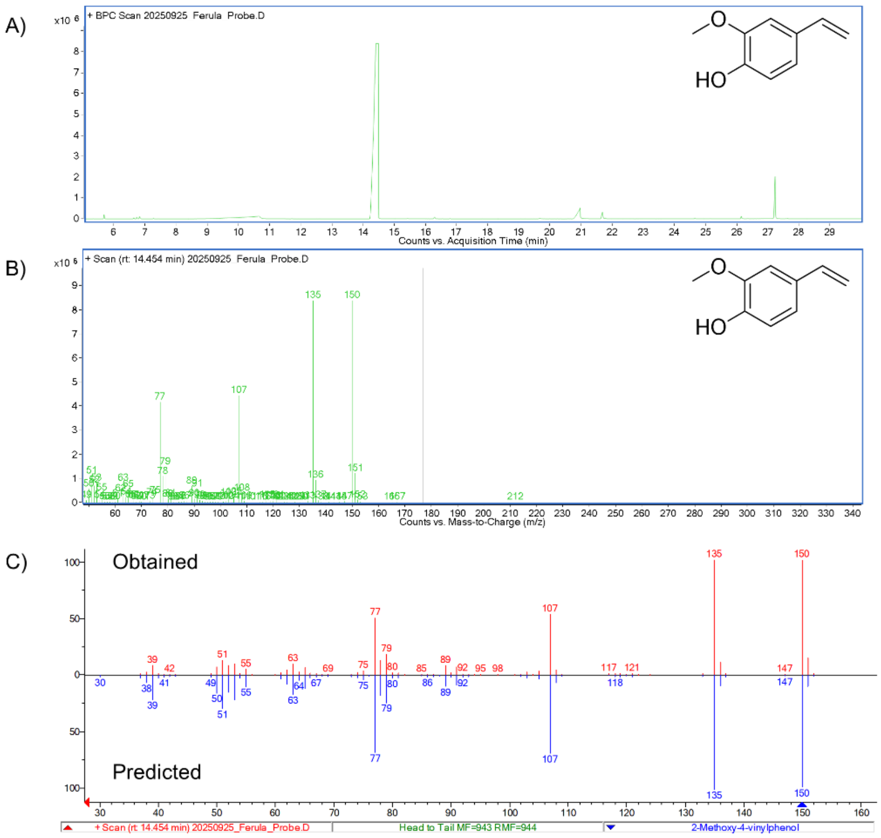

Figure S9. GC-MS of sample after 1 M reaction. A) GC chromatogram, B) MS chromatogram, C) comparison between the chromatogram obtained and the decay curve of 4-vinylguaiacol (m/z 150) predicted with NIST database.

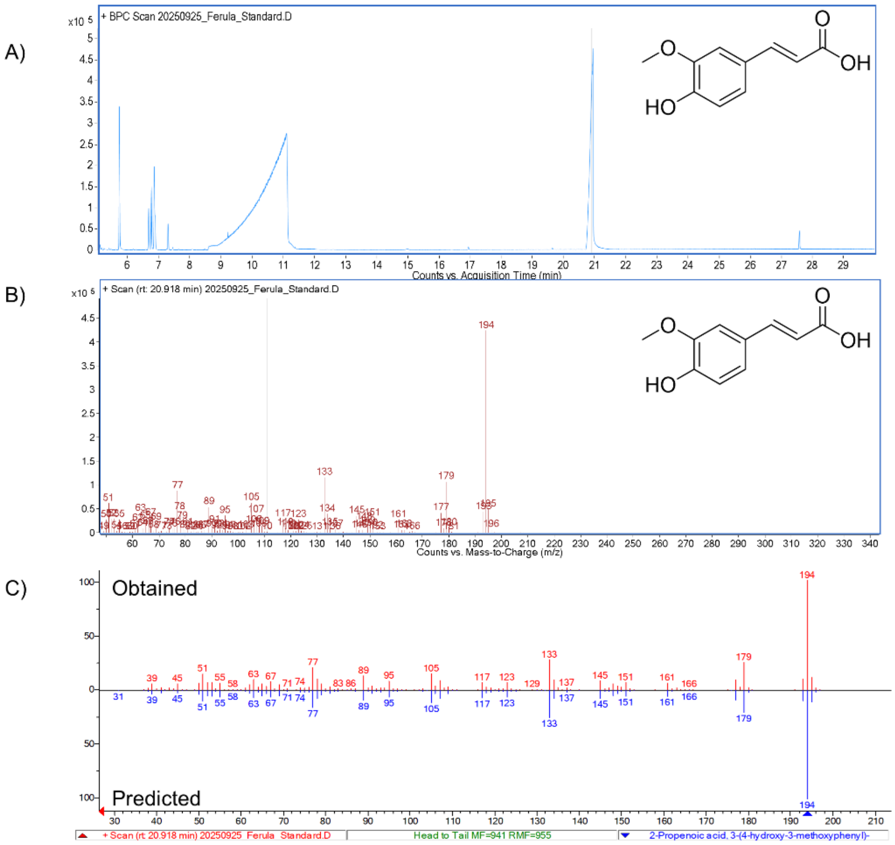

Figure S10. GC-MS of ferulic acid standard. A) GC chromatogram, B) MS chromatogram, C) comparison between the chromatogram obtained and the decay curve of ferulic acid (m/z 194) predicted with NIST database.

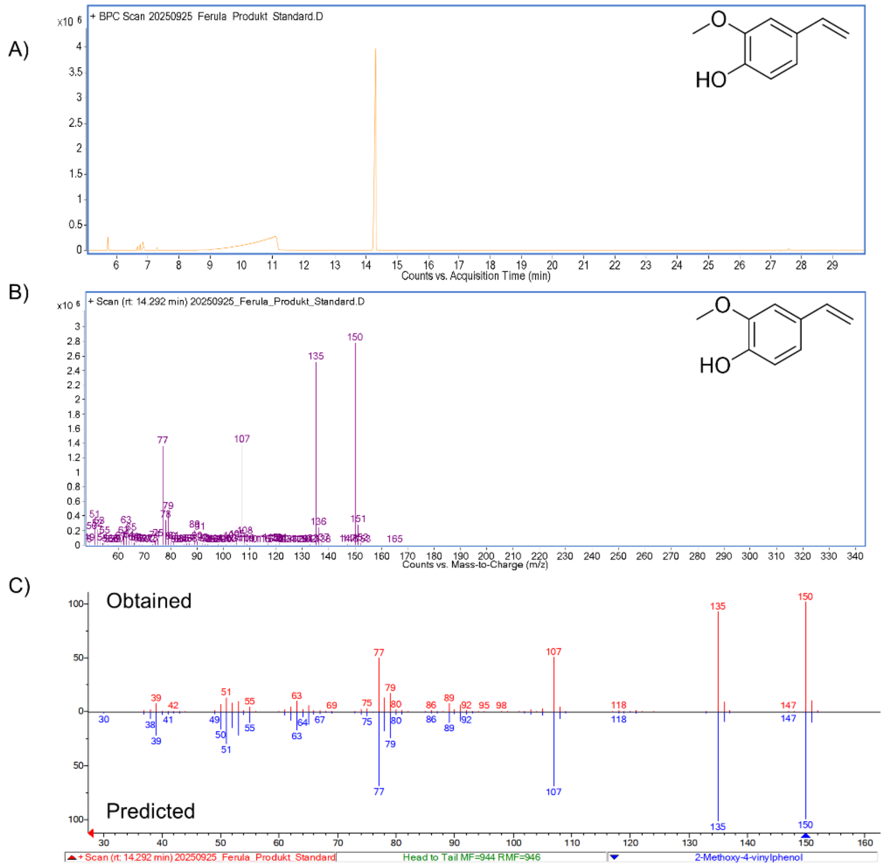

Figure S11. GC-MS of 4-vinyl guaiacol standard. A) GC chromatogram, B) MS chromatogram, C) comparison between the chromatogram obtained and the decay curve of 4-vinyl guaiacol (m/z 150) predicted with NIST database.
